# Anatomy packing with hierarchical segments: an algorithm for segmentation of pulmonary nodules in CT images

**DOI:** 10.1186/s12938-015-0043-3

**Published:** 2015-05-14

**Authors:** Chi-Hsuan Tsou, Kuo-Lung Lor, Yeun-Chung Chang, Chung-Ming Chen

**Affiliations:** Institute of Biomedical Engineering, College of Medicine and College of Engineering, National Taiwan University, Number 1, Section 1, Jen-Ai Road, Taipei 100, Taiwan; Department of Radiology, National Taiwan University College of Medicine, Number 7, Chung-Shan South Road, Taipei 100, Taiwan; Department of Medical Imaging, National Taiwan University Hospital, Number 7, Chung-Shan South Road, Taipei 100, Taiwan

**Keywords:** Lung CT images, Ground-glass nodule segmentation, Statistical region merging, Conditional random field, Hierarchical segmentation tree

## Abstract

**Background:**

This paper proposes a semantic segmentation algorithm that provides the spatial distribution patterns of pulmonary ground-glass nodules with solid portions in computed tomography (CT) images.

**Methods:**

The proposed segmentation algorithm, anatomy packing with hierarchical segments (APHS), performs pulmonary nodule segmentation and quantification in CT images. In particular, the APHS algorithm consists of two essential processes: hierarchical segmentation tree construction and anatomy packing. It constructs the hierarchical segmentation tree based on region attributes and local contour cues along the region boundaries. Each node of the tree corresponds to the soft boundary associated with a family of nested segmentations through different scales applied by a hierarchical segmentation operator that is used to decompose the image in a structurally coherent manner. The anatomy packing process detects and localizes individual object instances by optimizing a hierarchical conditional random field model. Ninety-two histopathologically confirmed pulmonary nodules were used to evaluate the performance of the proposed APHS algorithm. Further, a comparative study was conducted with two conventional multi-label image segmentation algorithms based on four assessment metrics: the modified Williams index, percentage statistic, overlapping ratio, and difference ratio.

**Results:**

Under the same framework, the proposed APHS algorithm was applied to two clinical applications: multi-label segmentation of nodules with a solid portion and surrounding tissues and pulmonary nodule segmentation. The results obtained indicate that the APHS-generated boundaries are comparable to manual delineations with a modified Williams index of 1.013. Further, the resulting segmentation of the APHS algorithm is also better than that achieved by two conventional multi-label image segmentation algorithms.

**Conclusions:**

The proposed two-level hierarchical segmentation algorithm effectively labelled the pulmonary nodule and its surrounding anatomic structures in lung CT images. This suggests that the generated multi-label structures can potentially serve as the basis for developing related clinical applications.

## Background

Pulmonary nodules, detected by volume computed tomography (VCT) scanning, are potential manifestations of lung cancer [1]. Nodule characterizations, including the shape complexity, volume size, and the percentage of ground-glass opacity (GGO) volume, have shown promise in helping with differential diagnosis and assessment of treatment response [2–8]. The nodule shape provides useful information for differentiating malignant from benign cases [4, 5] and several nodule shape characteristics are related to underlying pathology [2]. In clinical practice, volumetric measurement [3, 7] can accurately determine the nodule size to assess the growth of small nodules and calculate their volume doubling time (VDT). Mozley et al. [6] demonstrated that nodule growth rate has the potential to benefit medical practice. In addition, the percentage of solid versus ground-glass portions of the part-solid nodule is an important feature in terms of the relationship between the malignancy and the extent of the solid component [9, 10]. Automatic segmentation of pulmonary nodules is therefore well studied to provide reproducible quantitative measurements for diagnosis and avoid tedious manual labor [5, 11].

Current measurement methods for assessing the response of solid nodules to chemotherapy include one-dimensional (1D) longest in-slice dimension, two-dimensional (2D) area from longest in-slice and longest perpendicular dimension, and three-dimensional (3D) semiautomated volume [12, 13]. 1D and 2D CT measurements of small pulmonary nodules, although simple to implement, are limited by poor inter- and intra-observer variability [14] and complex nodule shapes [15]. In an effort to deal with the variety of nodule morphology and appearance properties [16] and to achieve high reproducibility [12], volumetric growth assessments have frequently been adopted for lung cancer screening and oncological therapy monitoring. For example, Gu et al. [17] developed a single-click ensemble segmentation approach with only one operator-selected seed point that facilitates processing of large numbers of cases. Although the utilization of these computer-aided segmentation algorithms were well validated for the segmentation of solid nodules, less research is done on subsolid nodules which actually show a higher malignancy rate than solid nodules [18, 19].

**Figure 1:**
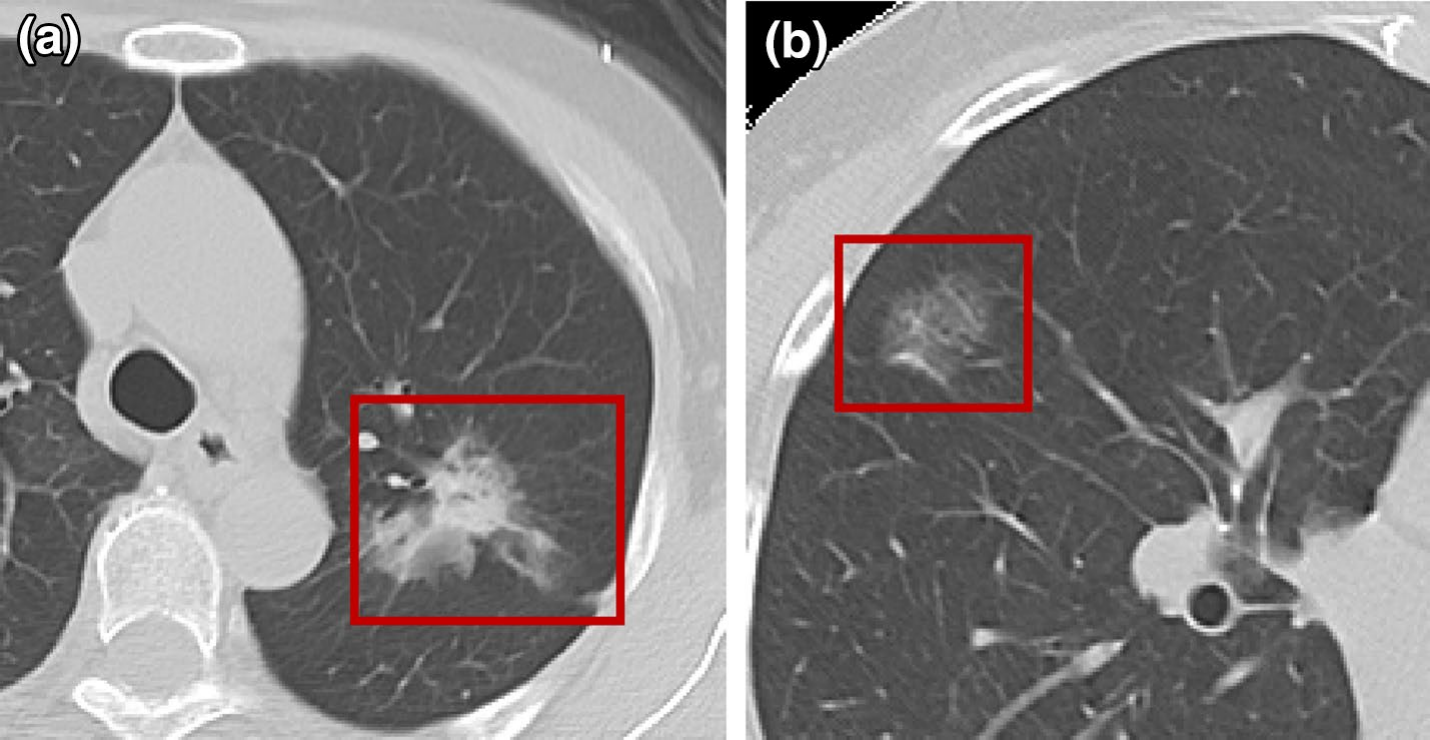
Pulmonary nodule on chest CT. **a** Thin-section CT with 3-mm collimation showing a 2.2-cm ground-glass dominant nodule at the upper left lobe. **b** Thin-section CT with 3-mm collimation showing a lobulated contour of the pure ground-glass nodule.

In subsolid nodules, the amount of informative features extracted from the pixels is limited. Therefore, the segmented nodules with strong heterogeneous texture show inconsistent boundaries, making these computer-aided segmentation algorithms less robust in quantitative analysis. The primary difficulty lies in estimating the proportion of ground-glass components in a nodule and its spatial distribution patterns. Figure 1 depicts a thin-section CT with 3-mm collimation showing a ground-glass dominant nodule and a pure ground-glass nodule. Pulmonary nodules, which we want to segment, are indicated by red boxes and have various appearances, implying various interpretations [20]. Further complicating the problem is the presence of numerous objects with various shapes and textures. Therefore, one cannot rely on visual assessment or thresholding for quantitative measurement.

We propose a compositional segmentation algorithm [called anatomy packing with hierarchical segments (APHS)] that overcomes these problems. The APHS algorithm consists of two essential processes: hierarchical segmentation tree construction and anatomy packing. In the first process, the perceptual grouping of the image can be defined as a tree of regions, ordered by inclusion. A joint framework is then utilized to extract the pool of segments from a hierarchical segmentation tree (HST). Each node of the tree corresponds to the soft boundary associated with a family of nested segmentations through different scales applied by a hierarchical segmentation operator (HSO) that is used to decompose the image in a structurally coherent manner. Based on the region attributes and local contour cues along the region boundaries, the HST is then represented as an ultrametric contour map (UCM) that represents an indexed hierarchy of regions as a soft boundary image. In the second process, anatomy packing localizes individual object instances by optimizing the hierarchy conditional random field (CRF) model based on the HST.

The remainder of this paper is organized as follows: we review related work on pulmonary nodule segmentation in “Related work”. “Methods” reviews the statistical region merging (SRM) method [21], details the concepts underling HST, and presents the proposed APHS algorithm. “Results” and “Discussion” discuss the results obtained from experiments conducted using APHS. “Conclusions” concludes this paper. (Note: an earlier version of this work was presented as a conference paper [22]. This journal version extends the previous work with more concrete examples of complete theories, experiments, and comparisons.)

## Related work

Instead of presenting a full review of pulmonary nodule segmentation algorithms, in this section, we discuss methods that are most relevant to our study. We begin by focusing on computerized segmentation of pulmonary nodules using freehand sketches or single click. We then review variational methods that have been successfully applied to many medical image processing problems. Finally, we discuss related work based on graph partitioning approaches applied to pulmonary nodule segmentation.

### Computerized segmentation of pulmonary nodules using single click or freehand sketches

Lung nodule segmentation plays a critical role in the development of computer-aided diagnosis (CADx) systems for lung cancer [5]. To improve the usability of segmentation algorithms, it is essential that computerized schemes with only a few incidents of manual user interaction be adopted in order to reduce inter- and intra-observer variability. Previous studies have demonstrated that these computerized schemes can address various types of pulmonary nodules, including high and low contrast nodules, nodules with vasculature attachment, and nodules in the close vicinity of the lung wall or diaphragm [17, 23, 24]. Many of these schemes adopted region growing with few seed points [17, 23] or break-and-repair strategies followed by freehand sketches [24].

Region growing is a technique in which the nodule boundary is delineated by identifying a seed point, calculating the connectivity of the points of the images to the seed point, and applying the halting criteria. The enhancement procedure is aimed at preserving the appearance of nodule margins and diminishing the background. Dehmeshki et al. [23] proposed a technique in which adaptive sphericity oriented contrast region growing is performed on a generated fuzzy connectivity map of the object of interest for nodule segmentation. However, the contrast between nodules and surrounding structures was changed due to different seed points. Gu et al. [17] proposed a CT-based single-click ensemble segmentation approach based on the use of multiple seed points with region growing, and then using a voting strategy to obtain the final tumor area.

Instead of utilizing single or multiple seed points, Qiang et al. [24] performed freehand sketching analysis to infer adaptive information (e.g., the mass center, the density, and the size) in regard to the nodule and then used principal curvature analysis and visibility test with convex constraint for nodule segmentation. As the preprocessing step of the computerized segmentation procedure, these methods repeat the segmentation multiple times using different multiple seeds that are proved to have low inter-observer variability and few operator interactions [17]. However, all pulmonary nodules detected on CT scans are classified into three groups [20]: solid nodule (homogenous soft-tissue attenuation), nonsolid nodule (hazy increased attenuation in the lung that does not obliterate the bronchial and vascular margins), and part-solid (consisting of both ground-glass and solid soft-tissue attenuation components). Therefore, modelling the intensity distributions of the nodule without taking local or global context information into account restricts the discriminating power of the computerized schemes.

### Segmentation models of lung CT images with context information

As discussed in the above algorithmic description, each step in the preceding algorithm is defined heuristically with many parameters. Translating these steps as variational formulations is therefore useful in reducing the number of parameters [25]. Previous study successfully applied level set method to medical imaging problems to unify supplementary terms with the energy function [26]. When segmenting the nodules from noise, inhomogeneities, and complex structures, it is useful to exploit the context information of targets in order to discriminate the boundaries of the nodule “head” [26]. To appropriately utilize low-level cues (e.g., intensity, texture, and contours), researchers have developed a strategy that integrates these information with prior knowledge [26, 27]. Farag et al. [26] proposed a method in which the image intensity statistical information is fused with the lung nodule shape model in a variational segmentation framework for lung nodule segmentation in CT images. For volumetric measurement, the shape-based level set method was performed slice-by-slice and the overall narrow band region was computed in 3D space. In addition, Tan et al. [27] integrated the marker-controlled watershed algorithm, geometric active contours, and Markov random field (MRF) to segment lung lesions on CT scans. In their method, the user only selects the region of interest around the nodule on one slice and then obtains information about nodule type (solid, part-solid, or nonsolid). The initial segmentation is generated using the marker-controlled watershed algorithm. According to the nodule type and initial nodule area, geometric active contours are then applied to refine the segmentation of solid nodules and MRF for GGO portions of part-solid nodules. However, variational methods have their limitations in terms of initial condition and generalized shape prior.

### Graph-based approaches for pulmonary nodule segmentation

The graph partitioning approaches in medical image analysis predominantly seek the best solution by minimizing an objective function defined over an undirected graph representing pairwise relationships between data elements (pixels, voxels, regions, or features) [28–30]. Wu et al. [29] proposed a voxel-based approach for parsing multi-class lung anatomies that uses a single feature set and classifier. The proposed approach uses a CRF model that incorporates texture features, gray-level, shape, and edge cues to improve the resulting segmentation. To further separate the contextual pulmonary structures of lung nodules, Wu et al. [29] presented a probability co-occurrence map that accurately detects whether a lung nodule is attached to any of the major lung anatomies. Conversely, Song et al. [30] introduced an additional interaction term in the energy function defined by the graph cut [31] and graph search [32] methods to tackle the problem when target surfaces or regions lack a clear edge with similar intensity distribution.

## Methods

**Figure 2:**
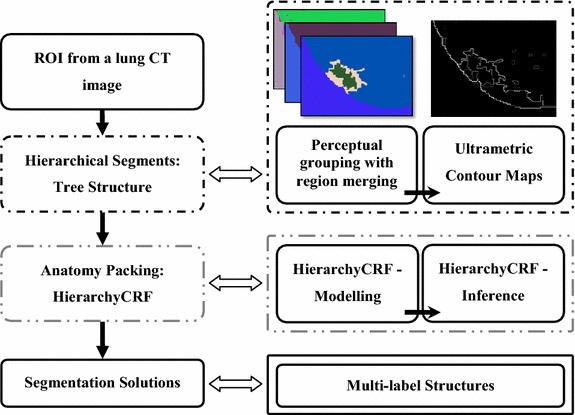
Steps in the proposed APHS algorithm. The *left column* outlines the major steps in the proposed algorithm. The *upper right*, *middle right*, and *bottom right diagrams* depict the HST, hierarchical CRF, and segmentation solutions processes, respectively.

The proposed APHS algorithm consists of two major steps: construction of hierarchical tree structures with pool segments, and anatomy packing. In the process of generating the pool of segments with similar texture, SRM [21] is used with the definition of the smallest region aggregation among pixels and a parameter setting for controlling the coarseness of the objects. The HST is then represented by an UCM [33]. In the second step, modelling and inference are performed in the CRF model based on the UCM [33]. The hierarchical CRF model can then be optimized by applying graph cut optimization [34]. Figure 2 gives a flowchart that outlines the steps comprising APHS. The details of each step are discussed in the ensuing sections.

### Hierarchical segments: tree structure

It is worth noting that pulmonary nodules can vary in terms of physical density of tissues, implying different appearances as a mixture of GGO and the solid part [20]. The effect of tissue inhomogeneity is therefore inevitable and has to be considered in the framework. According to Gestalt theory [35], perceptual grouping refers to the ability of human perception to transfer the collection of pixels of an image into a visually meaningful structure of regions and objects. For example, an observer delineates the nodule and its surrounding tissues by identifying physical objects and highlighting their boundaries with a certain level of detail. It postulates that if different observers perceive the same objects in a single CT image, the intersection of their drawing boundaries represents the finest level of details.

#### Perceptual grouping with region merging

The basic idea underlying SRM [21] is the formulation of image segmentation as an inference problem by seeking the optimized transformation of the collection of pixels into prominent structures. Under these conditions, the SRM [21] is applied with different scales to decompose the image in a structurally coherent manner. Based on a family of nested segmentations, the HST is generated by accumulating the local contour cues along the region boundaries. An HST is generated in two steps: perceptual grouping with region merging and UCM creation. The first step, perceptual grouping, is based on SRM [21] and combines regions or pixels, which are treated as elementary elements.

**Figure 3:**
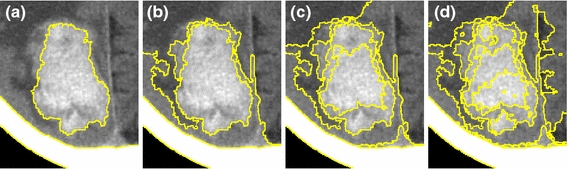
Segmentations of SRM on a synthetic grayscale image for various values of Q. **a** Q = 4, **b** Q = 16, **c** Q = 64, and **d** Q = 256. Q is a parameter that controls the statistical complexity of the image structures [21]. The regions found are *yellow-bordered*.

Let *S*_*i*_ be the set of couples of adjacent pixels and f(p, p′) = |I_p_ − I_p′_| that is to pick directly the pixel channel values (I_p_ and I_p′_) where pixel p and p′ belonging to image I. According to similarities between elementary elements, the couples of *S*_*i*_ are sorted in increasing order of *f* and this order is traversed only once. The merging predicate is then performed on measuring the couples of regions (*R*, *R′*). A family of nested segmentations of a synthetic grayscale image is demonstrated in Figure 3.

#### Creating an ultrametric contour map

In the second step, given the composite segmentations, the geometric structure of the image can be represented by a tree of regions, ordered by inclusion. Each node of the tree corresponds to an object at a certain scale of segmentation. Note that the entire scene is located at the root of the tree and the leaves are the finest details. The HST is therefore represented in terms of contours as an UCM [33, 36] that is an indexed hierarchy of regions as a soft boundary image. Let Ω ⊂ *R*^2^ be an image, *K*_0_ an initial contour from the initial partition of Ω and λ ∈ *R* a scale parameter. Based on multiple segmentation contours, the following characterizations of an HSO that assigns segmentation *K*_λ_ to the couple (*K*_0_, λ) are satisfied:

1 $$K_{\lambda } = K_{0} ,\quad \forall \lambda \, \le \, 0.$$

2 $$K_{\lambda } = \, \partial \varOmega ,\quad \forall \lambda \, \ge \, \lambda_{ 1} .$$

3 $$\lambda \le \lambda^{\prime } \Rightarrow K_{\lambda } \supseteq K_{{\lambda^{\prime } }} .$$

Relations (1) and (2) indicate that all inner contours vanish at finite scale. The principle of strong causality imposed by relation (3) ensures that localization of contours at different scales is preserved. Let γ be the ultrametric distance defined by a HSO. The ultrametric distances is defined by integrating local contour cues along the regions boundaries and combining this information with region attributes. The UCM generated by HSO is a single real-valued image and the application C(γ): *K*_0_ → [0, λ_1_] is given by

4 $${\text{C}}\left( \gamma \right)\left( \partial \right) \, = { \inf }\{ \lambda \in [0, \, \lambda_{ 1} ] \, | \, \partial \not\subset {\text{K}}_{\lambda } \} ,\quad \forall \partial \in K_{0} .$$

**Figure 4:**
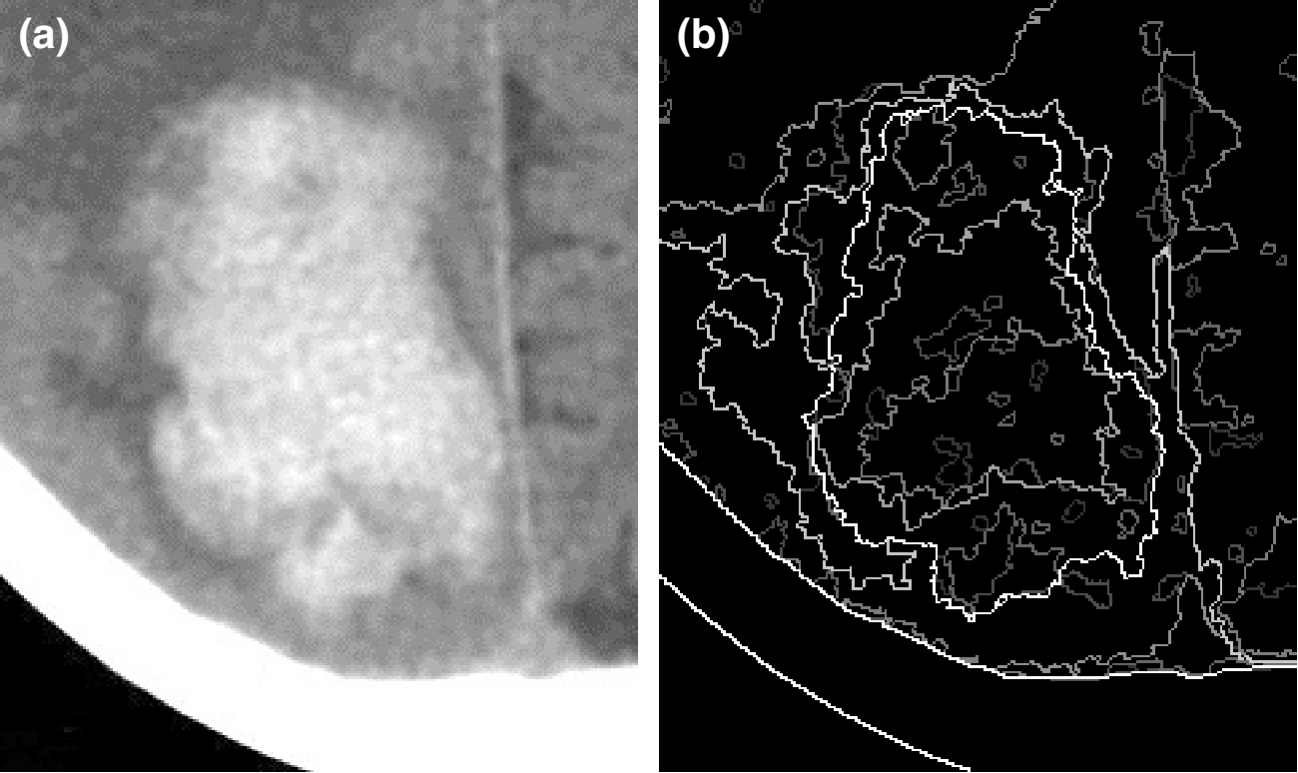
An illustration of the creation of a UCM. **a** A synthetic grayscale image. **b** The UCM.

The salience of contour ∂ is based on the number C(γ)(∂). A simple example of UCM, with a family of nested segmentations of a synthetic grayscale image, is presented in Figure 4.

### APHS for pulmonary nodule segmentation

Let $Q = (q_{1} ,{ \ldots },q_{n} ,{ \ldots },q_{N} )$ be a pool of segments that are all from the segmentation tree, and a vector $Y = (y_{1} ,{ \ldots },y_{n} ,{ \ldots },y_{N} )$ gives the label at each segment. The parameter $\theta$ can be represented by constructing histograms of gray values: $\theta = h(Q;Y)$. Parameters $Q$ and $\theta$ are used as observation functions to define the relationship between label $Y$ and observation features.

#### Anatomy packing: modelling and inference

The energy function, *E*, commonly used in the MRF model, is defined as a sum of unary and pairwise terms:

5 $$E(Y,\theta ,Q) = U(Y,\theta ,Q) + V(Y,Q).$$

The unary term, *U*, represents the cost of assigning the label $y_{i} > 0$ to a segment $q_{i}$, given the histogram model $\theta$, and can be written as:

6 $$U(Y,\theta ,Q) = \sum\limits_{n} { - \log h(q_{n} ;y_{n} )} .$$

Further, the pairwise term, as sum of boundary cost over the sets of adjacent pixel pairs (*i*, *j*), depends on the difference in pixel intensity:

7 $$V(Y,Q) = \gamma \sum\limits_{(i,j) \in C} {[y_{i} \ne y_{j} ]\exp - \beta (q_{i} - q_{j} )^{2} } .$$

where [.] represents the indicator function and *C* is the set of pairs of neighboring pixels. Note that pixels *i* and *j* belong to different segments that are assigned different nonzero semantic labels.

Given the HST from image *I*, these segments have to satisfy two constraints: the completeness constraint and the non-overlap constraint. First of all, the completeness constraint indicates that the label of each leaf segment is nonzero:

8 $$\forall p \in I,\quad \exists i:q_{i} \mathrel\backepsilon p,\quad y_{i} > 0.$$

Moreover, overlapping segments cannot take nonzero labels, according to the non-overlapping constraint that at most one of overlapping segments can be assigned 1 at the same time:

9 $$\forall i \ne j:q_{i} \cap q_{j} \ne \emptyset \Rightarrow y_{i} \cdot y_{j} = 0.$$

Putting this all together, the segmentation can be estimated as

10 $$\hat{Y} = \mathop {\arg \hbox{min} }\limits_{Y} E(Y,\theta ,Q).$$

According to Lempitsky et al. [34], in the case of a tree-based pool and K = 2, energy model (10) can be globally minimized subject to constraints (8) and (9) by graph cut [37, 38]. For the multi-class case, the alpha-expansion [38] can be used by performing a series of 2-class inferences for α sweeping the range 1 … K multiple times until convergence.

#### Segmentation by using anatomy packing

**Figure 5:**
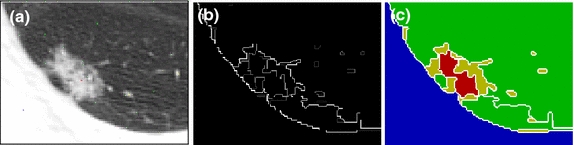
An illustration of the proposed APHS algorithm. **a** ROI of part-solid nodule in CT image. **b** A hierarchical edge map. **c** Resulting segmentation of the APHS algorithm (*green* parenchyma, *blue* wall, *red* solid nodule, and *yellow* GGO area and vessel).

This section discusses the details of the proposed APHS algorithm. The problem of delineating the pulmonary nodule is formulated as a multi-label graph partitioning. The HST is transformed into a corresponding graphical model by taking each segment as a graph node. The graph edges are then established to accumulate along the boundary between pool segments. The APHS algorithm can be summarized as follows: (1) Given a lung CT image with lung extraction, the region of interest (ROI) is defined by the user. (2) A family of nested segmentations of ROI is generated to set parameter Q ranging from one to 256 in powers of two. (3) The HST is then represented by using the UCM [33, 36]. (4) A set of K semantic labels, i.e., lung anatomies (e.g., parenchyma, lung wall, vessel, and nodule) is marked as the prior information. (5) A semantic segmentation framework modelling the weighting distribution of different lung tissues can be optimized by graph cut [37] or the alpha-expansion [38] algorithm for multi-label segmentation. An illustration of the proposed APHS algorithm is given in Figure 5.

### Image data and evaluation methods

Informed consent was waived for our retrospective study, which was approved by the local ethics committee. Ninety-two histopathologically confirmed lung nodules, receiving CT-guided lung biopsy or CT-guided localization for video-assisted thoracoscopic surgery (VATS) in our department, were used to evaluate the performance of the proposed algorithm. According to nodule consistency, there were 50 (50/92, 54.3%) and 42 (42/92, 45.7%) nodules with solid and non-solid or part-solid components, respectively. All CT studies were performed in full inspiration using either a 16-row multi-detector CT (MDCT) or a 64-row MDCT scanner (GE LightSpeed VCT; GE Healthcare, Milwaukee, WI, USA; Brilliance iCT; Philips Healthcare, Cleveland, OH, USA) with the following parameters: slice thickness 1.25 mm, standard reconstruction algorithm, 120 kVp.

The experiments was designed to assess the accuracy of the resulting segmentation, which was evaluated by comparing the computer-generated contours to four sets of manual delineations. The four sets of manual delineations were drawn by four graduate students majoring in biomedical engineering and trained to identify pulmonary nodules in CT images. The boundaries delineated by these four graduate students were further confirmed as acceptable by a thoracic radiologist (Y.C.C., with more than 20 years of experience). Further, the four sets of manual delineations were independently prepared by the four graduate students without any mutual interaction. Four metrics [39, 40], the modified Williams index, the percentage statistic, and the overlapping and difference ratios, were computed to provide quantitative comparisons between the four sets of manual delineations and computer-generated boundaries. Note that the four metrics were used to evaluate whether the computer-generated boundaries are comparable with manual delineation.

#### Modified Williams index

The first index was the modified Williams index [39], denoted by $WI\_C_{i}$, which is defined for each computer-generated boundary $C_{i}$, $1 \le k \le 4$:

11 $$WI\_C_{i} = \frac{{\frac{1}{n}\sum\nolimits_{j = 1}^{n} {{1 \mathord{\left/ {\vphantom {1 {AD_{{C_{i} ,O_{j} }} }}} \right. \kern-0pt} {AD_{{C_{i} ,O_{j} }} }}} }}{{\frac{2}{n(n - 2)}\sum\nolimits_{k = 1}^{n} {\sum\nolimits_{j = 1(j \ne k)}^{n} {{1 \mathord{\left/ {\vphantom {1 {AD_{{O_{k} ,O_{j} }} }}} \right. \kern-0pt} {AD_{{O_{k} ,O_{j} }} }}} } }}.$$

where $O_{j}$ denotes the *j*th set of manual delineations, *n* is the number of manual delineations for each nodule, and $AD_{S,T}$ is the average distance between the corresponding pair of boundaries in sets *S* and *T*. The distance between two compared boundaries $X \in S$ and $Y \in T$ can be defined as

12 $$AD(X,Y) = \frac{1}{2}\left\{ {\frac{1}{{N_{X} }}\sum\nolimits_{i = 1}^{{N_{X} }} {d(x_{i} ,Y)} + \frac{1}{{N_{Y} }}\sum\nolimits_{j = 1}^{{N_{Y} }} {d(y_{j} ,X)} } \right\}.$$

where $x_{i}$ and $y_{j}$ are the *i*th and *j*th points on boundaries $X$ and $Y,$ respectively. $N_{X}$ and $N_{Y}$ are the number of points on boundaries $X$ and $Y,$ respectively. $d(x_{i} ,Y) = \min_{j} \left\| {y_{j} - x_{i} } \right\|$ and $d(y_{i} ,X) = \min_{i} \left\| {x_{j} - y_{i} } \right\|.$ The numerator of the modified Williams index (11) represents the agreement between a set of computer-generated boundaries and the four sets of manual delineations. Under this definition, a large value for the numerator in (11) indicates a high degree of agreement between the set of computer-generated boundaries and manual delineations. On the other hand, the denominator in (11) quantifies the average degree of agreements between the manual delineations from different experts. The variation between the four sets of manual delineations is considerable when the denominator in (11) is small. Accordingly, if $WI\_C_{i}$ is greater than or equal to one, it suggests that the average distance between the computer-generated boundary $C_{i}$ and manual delineations is comparable to that between the manual delineations.

#### Percentage statistic

The second index was the percentage statistic $P\_C_{i}$ for each computer-generated boundary $C_{i}$, which is defined as the percentage by which the computer-to-observer distances are less than or equal to the corresponding maximum inter-observer distances. For each computer-generated boundary in $C_{i}$, *n* computer-to-observer distances are computed between the computer-generated boundary and the *n* manual delineations of the same nodule, respectively. One of the manual delineations of the same nodule serves as a reference boundary for each computer-to-observer distance. In addition, the maximum distance between the reference boundary and the remaining (*n* − 1) manual delineations indicates its corresponding maximum inter-observer distance.

#### Overlapping and difference ratios

In order to quantify the degree of matching between the areas of a computer-generated boundary and those of the corresponding average manual delineations of the same nodules, the third index, overlapping ratio, $OR\_C_{i}$ and the fourth index, difference ratio, $DR\_C_{i}$ for each $C_{i}$ were used. The overlapped region is the common area of the computer-generated boundary and the corresponding average manually delineated boundary, whereas the difference region is the area enclosed by either of these two boundaries, but not both. The overlapping ratio $OR\_C_{i}$ and the difference ratio $DR\_C_{i}$ are the mean ratios of the areas of the overlapped region and the difference region to the area of the corresponding average manually delineated boundary, respectively.

## Results

In this section, we discuss the application to multi-label segmentation of nodules with a solid portion and surrounding tissues and pulmonary nodule segmentation. Some qualitative and quantitative results from the experiments are also presented. In the semantic segmentation figures, different tissues are represented with specific colors, defined as green (parenchyma), blue (wall), red (solid part), and yellow (GGO components and vessel).

### Hierarchy example

**Figure 6:**
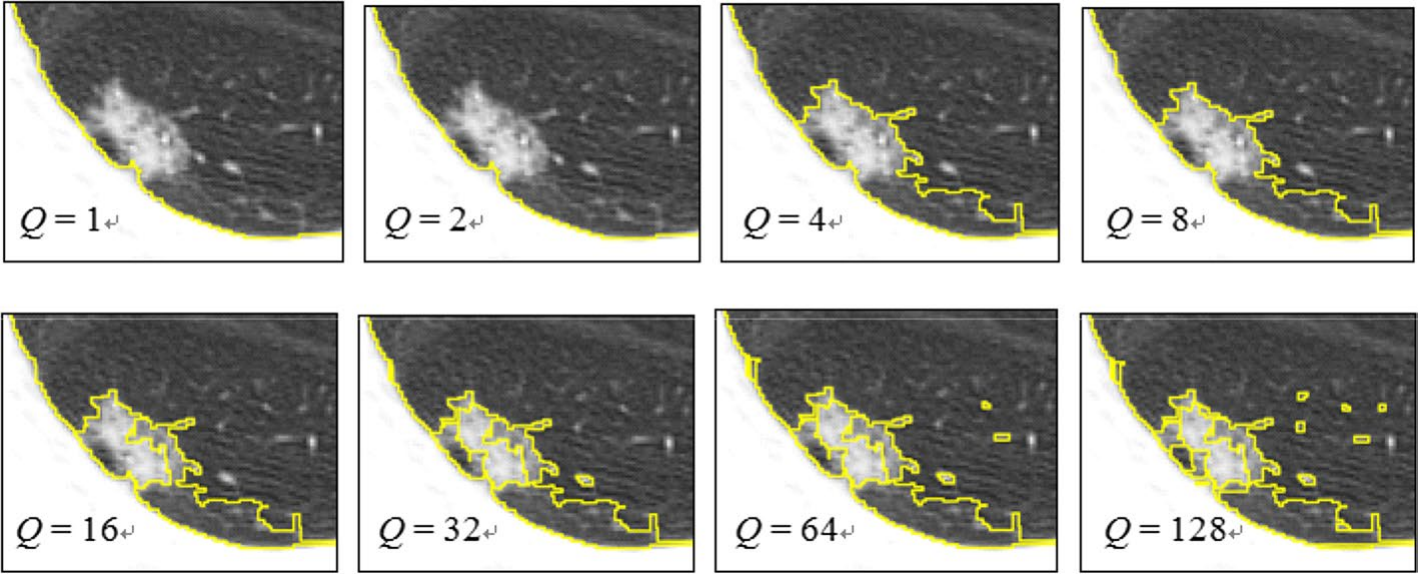
Segmentations of SRM on lung CT image, for various values of Q. Regions found are *yellow-bordered.*

Figure 6 depicts a family of nested segmentations of the pulmonary nodule in the CT image. In this perceptual grouping with the region merging process, at Q = 1, the boundary of the pleural wall is completely extracted. Further, both ground-glass and solid soft-tissue attenuation components are grouped into a single region. At Q = 32, the entire subregion with homogeneous soft-tissue attenuation is segmented while subregions, with hazy increased attenuation in the lung that does not obliterate the bronchial and vascular margins, are partly identified. At a finer level (Q = 128), the perceptual grouping with region merging process is able to capture the subregions of the ground-glass and solid soft-tissue. As Q increases, the statistical estimation task increases in accuracy. Note that tuning of parameter Q is carried out to control the coarseness of segmentation, which results in a level of perceptual details at each scale. In this paper, the optimal Q value was selected empirically for each individual case.

### Practical benefits of hierarchical CRF with pool segments

**Figure 7:**
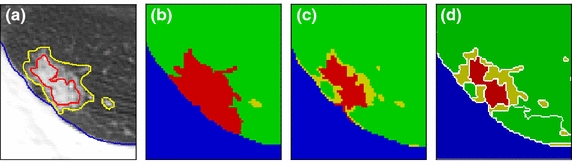
Comparison of various graph-based algorithms. **a** ROI of lung CT images with manual delineations. Demonstrations of the resulting segmentation: **b** random walks with restart [41], **c** nonparametric higher-order learning technique [42], and **d** the proposed algorithm. APHS appears to outperform the other approaches in terms of the quality of results. Note that marking roughly with semantic labels, i.e., lung anatomies (e.g., *green* parenchyma, *blue* lung wall, *yellow* vessel or GGO area, *red* nodule with solid part,* white* segment boundaries) is as the same prior information for each algorithm.

Figure 7 was demonstrated the feasibility of multi-label segmentation in APHS to identify different tissues. It can be observed in Figure 7 that GGO subregions with hazy increased attenuation are directly connected to other tissues and subregions with homogeneous soft-tissue attenuation. Figure 7 compares the resulting segmentation of the APHS with random walks with restart (RWR) [41], and nonparametric higher-order learning technique (NPHL) [42]. In Figure 7b, RWR [41] suffers over-segmentation in which the solid region includes GGO subregions and part of parenchyma. The steady-state probability of RWR [41] is defined as the relationship between the pixel and the seeds with the same label; thus, it is affected by the location of the seeds. In contrast to RWR [41], which defines the likelihood of a pixel as the average of all the steady-state probabilities, NPHL [42] considers not only the pairwise relationship between the pixels but also their corresponding regions in a multi-layer graph. Consequently, in this manner, local grouping cues were able to propagate into the whole image, it was less sensitive to user inputs, and gave the segmentation result with more detailed boundaries, as shown in Figure 7c. Figure 7d shows that the proposed algorithm can detect and localize the GGO subregions and its surrounding tissues.

### Quantitative results

To quantitatively evaluate the resulting segmentation of the proposed APHS algorithm, a comparative study with NPHL [42] and GrowCut [28] was conducted with the same user-specified seeds. NPHL [42] operates by recursively estimating from the likelihoods of pixels included in each region, generated by the unsupervised image segmentation algorithm. This motivated us to select NPHL [42] as a referential for comparison; furthermore, the source code of the implementation can be downloaded [42]. GrowCut [28] is based in cellular automation, which can treat pixel labelling process as growth and struggle for domination of the user-specified seed pixels. According to [28], GrowCut with the following advantages (multi-label segmentation, N-dimensional images processing, speed high enough for interactive segmentation) is capable of solving moderately hard segmentation problems and hence is referential for comparison.

To assess NPHL [42] and GrowCut [28], the aforementioned four assessment metrics were applied to evaluate the accuracy of its resulting segmentation with four sets of manual delineations. Table 1 compares the results obtained by NPHL [42] and GrowCut [28] to those obtained by the proposed APHS algorithm for the four metrics. The comparison between manual delineations and computer-generated boundaries (APHS, NPHL [42], and GrowCut [28]) shows a good agreement for the solid nodule data sets. However, the experiments with nonsolid nodules show inconsistent agreement, with the modified Williams indices (APHS 0.964, NPHL 0.859, GrowCut 0.976), the percentage statistics (APHS 72.8%, NPHL 55.9%, GrowCut 68.1%), the overlapping ratios (APHS 0.75, NPHL 0.64, GrowCut 0.7), and the difference ratios (APHS 0.25, NPHL 0.36, GrowCut 0.3). In general, it can be seen that the APHS-generated boundary sets are comparatively more stable and comparable to manual delineations than those of the NPHL [42] and GrowCut [28] algorithms.

**Table 1:** Performance evaluation

Algorithms	Modified Williams index	Percentage statistic (%)	Overlapping ratio	Difference ratio
APHS				
Solid	1.015	97.50 ± 1.67	0.858 ± 0.06	0.142 ± 0.06
Nonsolid	0.964	72.87 ± 4.72	0.747 ± 0.13	0.253 ± 0.13
All	1.013	86.81 ± 2.75	0.802 ± 0.09	0.198 ± 0.075
NPHL [42]				
Solid	1.038	93.34 ± 6.7	0.807 ± 0.16	0.193 ± 0.172
Nonsolid	0.859	55.85 ± 12.2	0.641 ± 0.11	0.359 ± 0.12
All	0.981	75.62 ± 4.16	0.723 ± 0.16	0.277 ± 011
Growcut [28]				
Solid	1.091	96.50 ± 1.88	0.841 ± 0.1	0.159 ± 0.09
Nonsolid	0.976	68.09 ± 7.96	0.696 ± 0.08	0.304 ± 0.11
All	1.081	80.52 ± 4.86	0.754 ± 0.12	0.246 ± 0.11

Comparison of APHS, NPHL [42] and Growcut [28] in evaluating the quality of the computer-generated boundaries with respect to the manually delineated boundaries using four metrics: the modified Williams index, percentage statistic, overlapping ratio and difference ratio.

To assess the stability of the APHS algorithm as a function of parameter Q, for each image, three different parameter Q ranges are used, the ranges of which are set to 1–32, 1–128, and 1–512, to generate different hierarchical segmentation trees at different scales. These three ranges are chosen because a Q less than 32 might remove desirable weak edges mistakenly and a Q larger than 512 tends to yield too many regions. The aforementioned assessment metrics (percentage statistics) was applied to evaluate the accuracy of the APHS algorithm with three different parameter ranges with four sets of manual delineations. Table 2 summarizes the means and standard deviations of the percentage statistics with respect to four observers for nodule boundaries derived by the APHS algorithm when Q = 1–32, 1–128, and 1–512.

**Table 2:** The means and standard deviations of the percentage statistic with respect to four observers for the nodule boundaries derived by the APHS algorithm when Q = 1–32, 1–128, and 1–512

	Q = 1–32 (%)	Q = 1–128 (%)	Q = 1–512 (%)
Percentage statistic			
Solid	83.34 ± 11.22	97.50 ± 1.67	99.12 ± 1.67
Nonsolid	12.23 ± 5.6	38.09 ± 7.27	53.62 ± 5.59
All	39.93 ± 6.74	51.96 ± 9.18	72.74 ± 6.99

Among Q = 1–32, 1–128, and 1–512, the performances for Q = 1–32 were worse than those for the other two in terms of the percentage statistics. This phenomenon may be imputed to the fact that, in the construction of the hierarchical segmentation tree, the desirable weak edges are more likely to be eliminated mistakenly by using Q = 1–32 than by using the other two, which leads to a larger deviation from the desired boundary. The percentage statistics validates that the nodule boundaries derived by the APHS algorithm are comparable to the manually delineated boundaries when Q = 1–512. In practical implementation, a small Q (e.g., Q = 1–32) is suggested to be used first if no apparent weak edges exist in the nodule boundary (e.g., solid nodule), which tends to result in a smaller number of regions per nodule. However, a more conservative choice of Q would be Q = 1–512, which with a higher probability to get more details compatible with the perceptual organization of the image.

## Discussion

In this section, we compare our approach to that of the multi-label image segmentation algorithm on the clinical dataset and investigate some of the trade-offs of our approach.

Approaches based on the concept of decision forests [43] can be used to tackle common learning tasks such as classification, regression, density estimation, manifold learning, semi-supervised learning, and active learning. In these hierarchical tree frameworks, the tests associated with each split node and the decision-making predictors associated with each leaf are two key components that enable a decision tree to function properly, such that the input data are presented as high-dimensional image representations. Konukoglu et al. [44] extracted the high-dimensional representation of images based on appearance features without any prior assumptions on their individual relevancy or their compactness. Their neighborhood approximation forests algorithm can handle neighborhood structures induced by the application-specific distance; therefore, the prediction procedure focuses on the co-occurrences of the test image and all database images that reach the same leaves of the trees. Criminisi et al. [45] achieved anatomy detection and localization in CT scans by maximizing the confidence of the predictions over the position of all organs of interest based on regression forests. In contrast to these methods, our HST is constructed based on a UCM by using region attributes and local contour cues along the region boundaries for each single case. As shown by Socher et al. [46], this facilitates the understanding and classifying of scene images. Here, we detect and localize individual objects by applying a hierarchical CRF on the HST; thereby achieving simultaneous GGO estimation and its surrounding tissues identification as shown in Figure 7.

In clinical practice, the region-based scheme [40] has been shown to be more effective in medical imaging. The advantage of tessellating medical images into regions is twofold. Firstly, the regional structure can provide sufficient informative features extracted from the pixels within the regions. Secondly, the search space spanned by regions is far less than that spanned by pixels. Consequently, finding the optimal segmentation solution by regions is more efficient than doing so by pixels. Compared to pixel-based approach [41], the prominent region structure offers less negative influences by surrounding tissues. In addition, the computational search space contains considerably fewer prominent data structures in regions, and hence performs more efficiently. Our algorithm is less sensitive to user inputs and yet gives high-quality segmentation results.

## Conclusions

In this paper, we proposed a region-based graph cut algorithm, called the APHS algorithm, to tackle a major challenge in quantitative measurements for diagnosis: estimating the proportion of GGO and identifying its surrounding tissues simultaneously. We formulated the problem of pulmonary nodule segmentation as a multi-label function, with the following results: (1) an anatomy packing process that can be optimized by utilizing a generic optimization algorithm, specifically, graph cut, which is frequently used in the computer vision community and (2) a relationship between GGO and the solid part that can be detected and localized according to gradient information.

A clinical database of nodules that underwent VATS was used to evaluate the performance of the proposed APHS algorithm and its accuracy and efficiency evaluated using four assessment metrics: “modified Williams index”, “percentage statistics”, “overlapping ratio”, and “difference ratio”. Further, its performance was compared with two conventional multi-label approaches and was shown to be superior in dealing with highly heterogeneous tissue content. The boundaries generated by the APHS algorithm can potentially serve as the components of CADx or other related clinical applications. For future considerations, robust higher-order potentials will be investigated in order to enforce label consistency in pool segments [47]. Other important tasks that will also be considered are automation of the initialization process and expansion to 3D volume measurements with further performance evaluations.

## Abbreviations

GGNs: ground-glass nodules
CT: computed tomography
APHS: anatomy packing with hierarchical segments
VCT: volume computed tomography
GGO: ground-glass opacity
VDT: volume doubling time
1D: one-dimensional
2D: two-dimensional
3D: three-dimensional
HST: hierarchical segmentation tree
HSO: hierarchical segmentation operator
CRF: conditional random field
SRM: statistical region merging
CADx: computer-aided diagnosis
MRF: Markov random field
UCM: ultrametric contour map
ROI: region of interest
VATS: video-assisted thoracoscopic surgery
MDCT: multi-detector CT
RWR: random walks with restart
NPHL: nonparametric higher-order learning technique.
